# Detection of Magnetomechanical Effect in Structural Steel Using GMR 2nd Order Gradiometer Based Sensors

**DOI:** 10.3390/s19194147

**Published:** 2019-09-25

**Authors:** Carmela Bonavolontà, Massimo Valentino, Francesco Penta, Carmine Granata, Berardo Ruggiero, Paolo Silvestrini, Antonio Vettoliere

**Affiliations:** 1 INFN—Istituto Nazionale di Fisica Nucleare, sezione di Napoli, 80126 Napoli, Italy; bonavolo@na.infn.it (C.B.); m.valentino@isasi.cnr.it (M.V.); 2 Istituto di Scienze Applicate e Sistemi Intelligenti del CNR, 80078 Pozzuoli, Italy; carmine.granata@cnr.it (C.G.); b.ruggiero@isasi.cnr.it (B.R.); paolo.silvestrini@unicampania.it (P.S.); 3 Department of Industrial Engineering, University of Naples “Federico II”, 80125 Naples, Italy; francesco.penta@unina.it; 4 Department of Mathematics and Physics, University of Campania “L. Vanvitelli”, 81100 Caserta, Italy

**Keywords:** magneto-mechanical effects, non-destructive testing of materials, GMR devices

## Abstract

The magneto-mechanical behaviour of structural steel specimens stressed up to the plastic deformation stage was investigated using a 2nd order gradiometer based on Giant Magneto Resistive (GMR) sensors. The correlation between the gradient of the magnetization and the dislocation density before the crack initiation inside the test material was reported. The capability of the GMR scanning sensor to detect the residual magnetization due to the tensile stress with a non-invasive technique was demonstrated.

## 1. Introduction

Since 1842, it is well known that the effect called inverse Joule, Villari or piezo-magnetism consists in the changing of magnetic properties of a metallic material when it is stressed or strained. In particular, the susceptibility and the permeability of the material can change because an anisotropic deformation of the crystal lattice is produced by applying the strain. The materials in which it is possible to observe this effect are mainly 3D metal, alloy, intermetallic compounds and in 4f metals (lanthanide compounds).

Depending on the type of strain, the elastic, plastic or cyclic different material parameters can change through, such as, the resultant of the magnetic moment, the magnetic hysteresis loop of the material, and the magnetic flux dispersion with nucleation of fatigue cracks, respectively. In all the above situations the physical properties, for example magnetic hysteresis, Barkhausen noise and coercitivity, could change progressively when the fatigue process evolves from its beginning stage until the material finally fractures. 

When plastic deformation occurs, the first relevant structural alteration is represented by the increasing dislocation density. During the deformation process, at first the dislocation density surges rapidly with hardness, then it saturates and the hardness reaches a maximum value that remains constant even if further deformation occurs. The dislocations produced into the material by the plastic deformation interact and multiply through the crystal lattice, and as a consequence they interact with the magnetic domain wall influencing the magnetic structure and properties of the ferromagnetic materials. The plastic strains are non-linearly coupled with magnetic properties in the case of steels [1]. The experimental tests carried out both on carbon and electrical steels have highlighted that the influence exerted by plastic strains on material magnetic responses is stronger at the early stages of the deformation process and generally leads to a degradation of the material behaviour, since magnetic losses increased and permeability decreased. In addition, significant changes of magnetostrictive behaviour have also been observed [2]. This strong coupling is due to some mechanisms acting on the local scale. More specifically, the interactions between the magnetic microstructure (magnetic domains and walls) and the mechanical microstructure (dislocations, grains, stress fields) are the basis of the phenomenon [1,3]. The common accepted approach is considering microstructural defects generated by the plastic slips process as pinning centres for domain walls motion [4].

Currently, to guarantee the safety of large structures, such as buildings and bridges, it is necessary to detect in advance the damage produced during the life cycle of the metallic materials. In particular, to prevent crack initiation, it is useful to detect the starting stage of deformation when the dislocation density begins to increase. To this aim, the correlation between the strain and crack detection has been taken into account. In other words, several non-destructive methods based on the magneto-mechanical effect are able to successfully detect the mechanical stress [5,6,7,8,9,10,11,12,13]. One of the techniques widely used is centred on the detection of the strain effects in the magnetic hysteresis loop of the material. Unfortunately, this technique has some disadvantages: First, the restriction regarding the compatibility between the set-up geometry and shape of the specimens; second, this type of investigation method only allows the average magnetic properties, but it is not able to detect damage at a very early stage, neither its position nor the concentration of stress.

To overcome these limitations, other non-destructive testing methods could be used to detect magneto-mechanical effects in metallic alloys, such as the application of several magnetic sensors i.e., Hall probes, the superconducting quantum interference device (SQUIDs), fluxgate and the giant magneto-resistance (GMR) [14,15,16,17,18,19]. Using magnetic sensors characterized by the high magnetic sensitivity and spatial resolution, it is possible to achieve a correct localization of the plastic deformation on the sample surface. In this work, a non-destructive method based on the GMR magnetic sensor is applied to detect plastic deformation in ferromagnetic specimens. As at the early plastic stage, a fast and significant increase of the dislocation density occurs, the relative magnetic field signal is large enough for GMR sensors to detect changes of the dislocation density by means of variation of the magnetic signal. 

In this work, to improve the signal to noise ratio, a GMR second order gradiometer was designed and used to monitor magneto-mechanical behaviour in ferromagnetic specimens under a mechanical load. The aim of this work is to demonstrate that an alternative non-destructive method based on GMR sensors allows the correlation of the magnetization signal of the sample both with its mechanical behaviour and the localization of crack initiation, the latter being due to the imaging of the magnetic field.

## 2. Experimental Set Up and Sample Description

In this work, a dog-bone shaped sample, 6 mm thick, made of mild steel (Fe360) was analysed. This sample shape gives the possibility of investigating the effect of plastic deformation represented by surface deformations called slip bands. 

**Figure 1:**
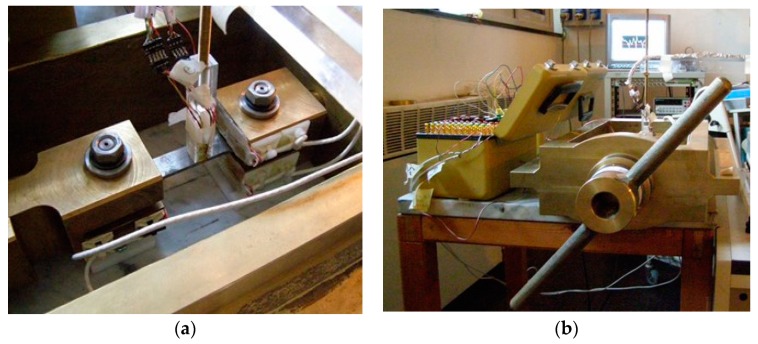
(**a**) Top view of the testing fixture and 2nd order gradiometer assembled on the dog-bone Fe360 at 0.2 mm from sample surface; (**b**) the side view of the testing fixture and details of the brass manual loading knob.

The testing sample was demagnetized in a long solenoid with the aim of cutting down the potential presence of any macroscopic residual magnetization. The testing fixture used to the applied load cycles to the sample is made of commercial brass, a non-magnetic material, which does not produce any detectable magnetic contribution that could modify the magnetic field detected nearby the sensors and the sample (see Figure 1).

**Figure 2:**
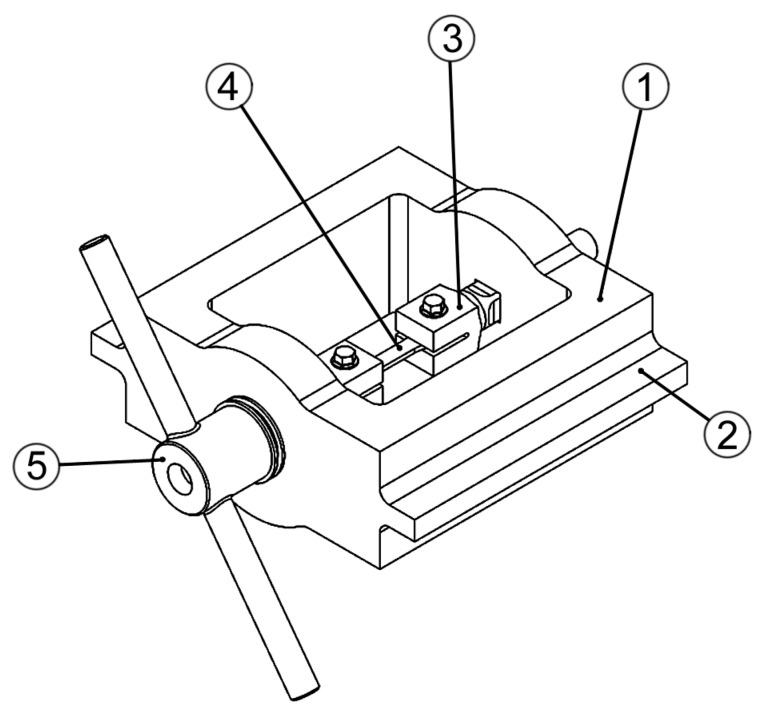
Testing fixture: (1) load frame, (2) stiffener, (3) forks with threaded ends, (4) specimen, (5) loading nut.

The fixture was essentially composed of a load frame consisting of two stout columns, stiffened by lateral ribs, and two crossheads having cross-sections gradually enlarged in their central region. In the mid-span sections, two holes with squared shape were made to locate the stems of the forks where the specimen was fastened by means of a couple of bolts. The testing load was generated by tightening one of the nuts screwed on the forks’ ends. The shape of the fork stems’ cross-section and holes in the cross-heads was adopted to avoid that the specimens were subjected to a parasitic twist during nut tightening. More details on the adopted fixture are given in Figure 2.

**Figure 3:**
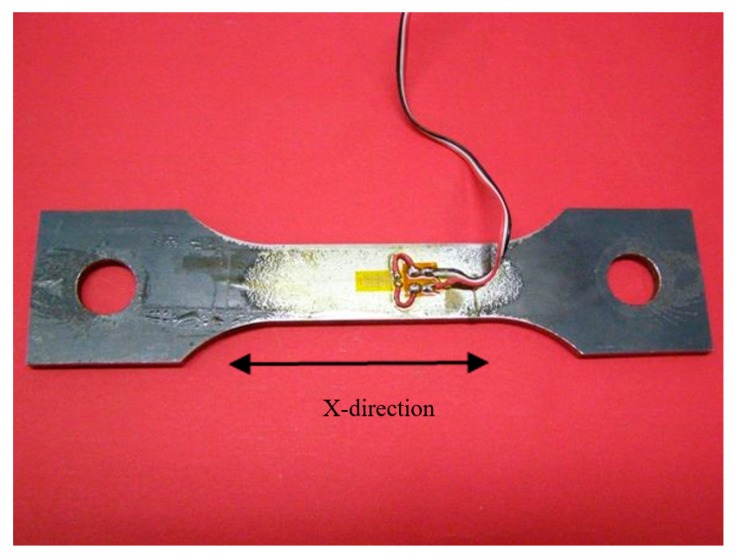
The dog-bone shape geometry of the Fe360 sample used for the mechanical test. In the picture the electrical wires are connected to the strain gage sensor glued on the yellow area as shown in the photo.

Firstly, the sample was demagnetized and then deformed plastically with a stress cycle ranging from 0 to 20 kN and successively, the loading cycle increased up to 40 kN. On each threaded bar, three electric strain gages were installed to measure and control the load. The signals of these strain sensors were calibrated by a universal servo hydraulic testing machine equipped with a load cell. The tensile stress was applied along the x-direction and measured by the strain gauges glued on both sides of the samples (see Figure 3). 

## 3. GMR Second Order Gradiometer Used to Detect the Mechanical Strain

A giant magneto resistive (GMR) sensor is based on the giant magneto resistive effect, discovered since 1988 as a large change in electrical resistance (from 10 % to 20 %) that occurs when thin, stacked layers of ferromagnetic and non-magnetic materials are exposed to a magnetic field [10].

**Figure 4:**
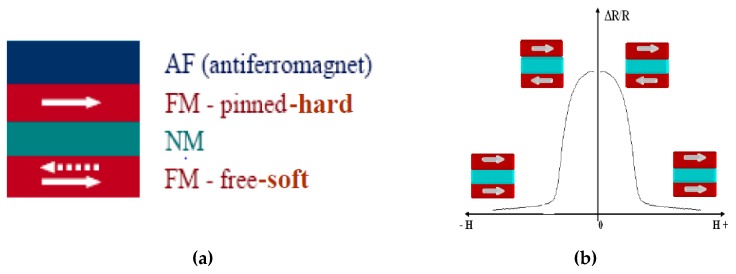
(**a**) Stacking sequence of the magnetic and non-magnetic layers in a giant magneto resistive (GMR) sensor, the dotted arrow stands for the antiparallel magnetization axis with respect to the hard layer; (**b**) the electrical resistance variation ∆R/R as function of an external applied magnetic field H.

A single GMR sensor essentially consists of two magnetic layers, one being magnetically hard while the other is soft, separated by a non-magnetic spacer with a total thickness of approximately 10 nm. The magnetization of the soft magnetic layer (detector) changes its direction under an applied external magnetic field with respect to the fixed magnetization of the hard layer, pinned by an antiferromagnetic layer (as shown in Figure 4a). The electrical resistance of GMR sensor depends on the relative orientation of the magnetic moment of the magnetic layers. In particular, the signal of the GMR is proportional to the cosine of the angle between the magnetization of the soft and hard layers. The electric resistance is minimal if the magnetization axis of the soft layer is parallel to the hard layer and is maximum if the two magnetization axes are anti-parallel (as shown in Figure 4b). Then, the GMR sensors are generally used in magnetic field sensing applications, detecting the component of the uniform magnetic field parallel to its uniaxial sensitivity axis. The GMR sensors offer a high thermal stability, a low power consumption, a wide linear range of operation, DC to 1 MHz frequency response and a small size. 

**Figure 5:**
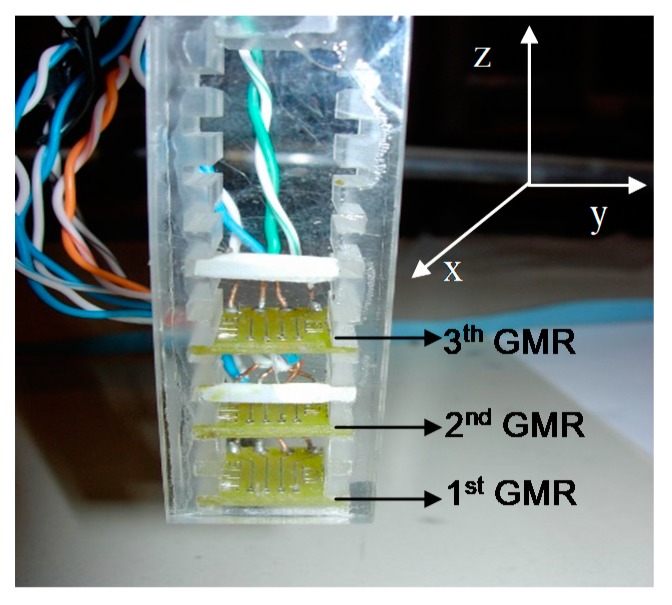
GMR 2nd order electronic gradiometer. It detects the in plane (y-axis) magnetic field component reducing the environmental interference.

In this work, a second order electronic gradiometer configuration for non-destructive inspection of materials is presented. The device was based on three on-chip GMR sensors fabricated by NVE (NonVolatile Electronics Inc., model AA005-02, Eden Praire, MN, USA), and arranged into a plexiglass holder to form a second order electronic gradiometer, as shown in Figure 5.

The GMR on-chip sensor is a magnetometer configured as a Wheatstone bridge to provide temperature compensation, and it is characterized by two flux concentrators that provide a directionally sensitive output signal. Since these devices are sensitive only in the direction of the supplied current plane, they record the same output for magnetic fields that have the positive or negative direction with respect to the GMR sensitivity axis. The three GMR-sensors’ magnetometer were vertically aligned with a baseline of 10 mm, and each sensor was biased with a constant supply voltage of 9 V. The 1st GMR sensor that was close to the sample (lift-off of 0.5 mm) was the detector, while the other two GMRs (2nd and 3rd) provided references to subtract and reduce the environmental interference. The 2nd order gradiometer based on GMR sensor improves the ability of the GMR magnetometer to reject the environmental interference [16]. 

To demonstrate the reproducibility of the GMR measurements, the stability of the sensors’ response and the influence of the environmental magnetic field, mainly due to electric power line at 50 Hz, a preliminary study was carried out. 

**Figure 6:**
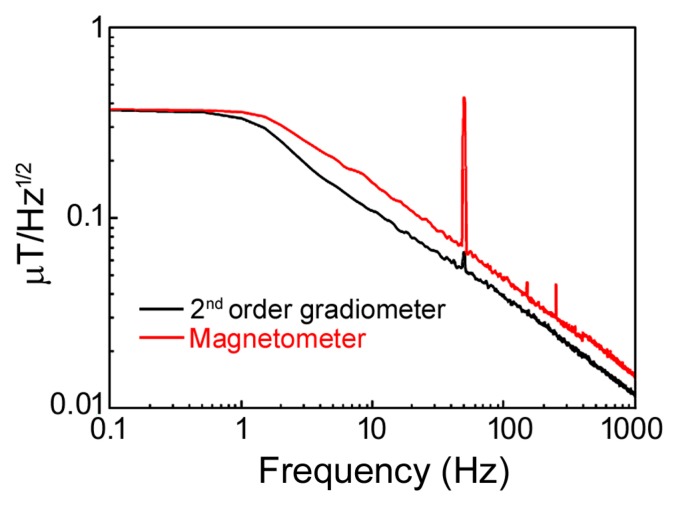
Comparison between the 2nd order GMR gradiometer (black line) and GMR magnetometer spectral noise in a harsh environment.

In Figure 6, the comparison between the spectral noise of a GMR magnetometer and the realized gradiometer at room temperature in a harsh environment is shown. It could be noted that the second order gradiometer is able to successfully reduce the environmental noise, especially at 50 Hz and their multiple harmonic frequencies. Then, the signal intensity due to the power line at 50 Hz measured by the gradiometer probe decreases 6.5 times with respect to the magnetometer, while the spectral lines at higher frequencies are not visible. This means that the gradiometer reduces the environmental interference in a wide frequency range (from 1 Hz up to 1 kHz).

**Figure 7:**
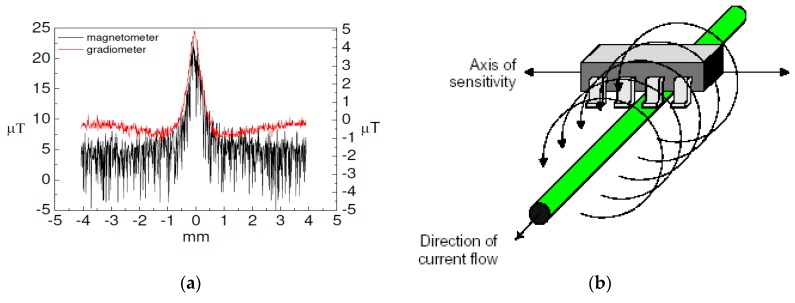
(**a**) Comparison between the magnetic field, detected by the GMR magnetometer (black line, scale on the left y-axis) and the 2nd order GMR gradiometer (red line, scale on the right y-axis), produced by an electric wire positioned at 0 mm on the x-axis, and the left and the right y-axis is related to the output signal measured by the magnetometer and gradiometer, respectively; (**b**) the sketch of the wire (with diameter of 0.2 mm) and its magnetic field (circular arrows). The direction of the magnetic field is along the GMR axis of sensitivity.

A calibration of the GMR magnetometer and gradiometer configuration to study the effect due to the sensor intrinsic hysteresis was carried out. Using a motorized scanning micrometric stage, the GMR sensor was moved over the electric wire (Figure 7b) from right to left and vice versa, and the magnetic field signal was recorded. The scanning lines (as in Figure 7 (black line)) overlapped within the signal ripple. The same procedure was done using the GMR gradiometer, and the curves obtained (as in Figure 7 (red line)) can be superimposed with an uncertainty of less than 3%. Therefore, the magnetometer GMR intrinsic hysteresis in a gradiometer configuration could be estimated as at most 3% of the measured signal when the magnetic field variation is of the order of μT.

Moreover, Figure 7a shows the outputs of the single GMR sensor and the gradiometer in correspondence with the magnetic field source induced by the biased electrical wire. This demonstrates that the gradiometer configuration reduces the environmental interference which improves the quality signal of the measurement.

It is noted that the magnetic field measured by the single magnetometer has a ripple larger than the gradiometer. This means that a reduction of the noise in an unshielded environment could be obtained using a second order gradiometer configuration. Moreover, the gradiometer output was affected by a five times reduction of the signal amplitude as confirmed by the values at maximum (x = 0).

## 4. Results and Discussion

**Figure 8:**
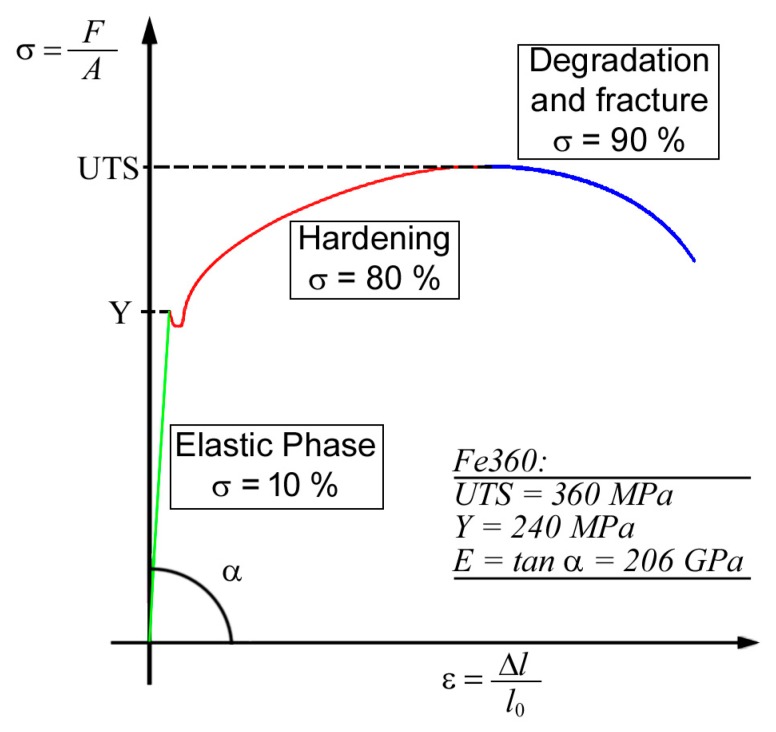
Schematic view of mechanical load- deformation of a metallic material.

The graph in Figure 8 shows the changing load as a function of deformation during a tensile test. The hardening phase where significant changes in dislocation density occur are highlighted. Furthermore, it is recalled that the fatigue life of a component is composed of three different stages. The first one ends at 10% of the fatigue life and presents consistent changes in dislocation density. The second lasts until 80% of the fatigue life is reached and it is characterized by the formation of permanent slip bands that could cause cracks. The third stage involves the formation of fatigue cracks and the final failure. 

Generally, it is difficult to detect crack nucleation before 90% of the fatigue life, even if it occurs earlier in the fatigue life cycle and propagates into the specimens very rapidly up to the catastrophic failure of the material. For this reason, it is necessary to detect in advance the fatigue crack initiation to guarantee the safety of large structures made of metallic alloys. Then, one of the NDE useful techniques used to detect the first and third stages of fatigue is represented by magnetic field sensors, such as GMR sensors, which are able to detect the magnetic field variation produced by the mechanical structural changes. 

**Figure 9:**
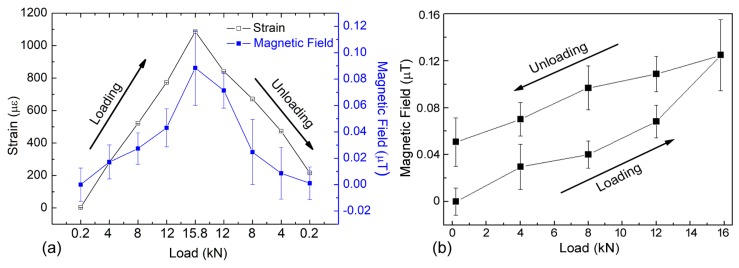
(**a**) Comparison between the strain gage signal (left scale) and the magnetic field (right scale) detected by GMR gradiometer during the load and unload test from 0.2 kN to 15.8 kN; (**b**) detailed of the magnetic field detected by GMR gradiometer during the loading cycle up to 15.8 kN in its properly x-axis scale.

In this work, the second order gradiometer based on GMR on-chip sensors was used to achieve this aim. In Figure 9a, the magnetic field due to the applied load is depicted in comparison with the produced strain. It is noted that the two curves have the same behaviour. When the strain increases the gradiometer signal does the same, and in the same way a decreasing of the magnetic field corresponds to a strain reduction. Therefore, there is proportionality between the strain effects and the magnetic field produced by the structural changes of the sample. 

**Figure 10:**
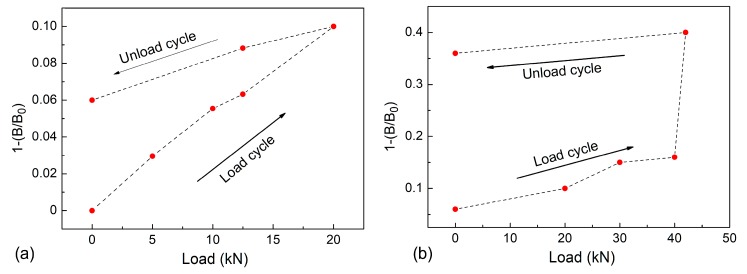
Normalized magnetic field signal during the load cycle up to 20 kN (**a**) and 40 kN (**b**). B is the residual magnetic field measured by the gradiometer while B_0_ is the magnetization field (generally, of the order of few μT) measured before the stress test. In both graphs, a hysteretic behaviour is recorded.

Figure 9b shows the behaviour of the magnetic hysteresis due to the loading cycles. Due to the gradiometer sensitivity, it is possible to understand the magnetic hysteresis produced into the sample as a function of loading effect. In Figure 10, the normalised signal detected by the second order gradiometer is reported in correspondence to the load cycle. In particular, in Figure 10a the load cycle increases up to 20 kN and a narrow hysteresis is detected. On the other hand, when the load cycle reaches 40 kN of the maximum loading, the hysteresis is greater and wider than that revealed when the maximum cycle is lower (20 kN), see Figure 10b. Then, it was verified that for loads below 10 kN, the response of the GMR gradiometer did not show magnetic hysteresis produced by the material. Moreover, from Figure 10, the starting value of the cycle at 40 kN corresponds to the final value of the cycle at 20 kN, despite the fact that it was not measured on the same day. This result demonstrates that the sample magnetization has been modified as a consequence of the loading cycle up to 20 kN, so it is reasonable to infer that the measured hysteresis is mainly due to the magnetic effects in the material and not to the hysteresis of the gradiometer.

Furthermore, the experimental results in Figure 10 demonstrate the capability of the GMR second order gradiometer to detect the effect of the load cycle in such materials. Furthermore, at the same time, the possibility of detecting the deformation degree described by the magnetic hysteresis increases when the material undergoes high cyclic loading. 

**Figure 11:**
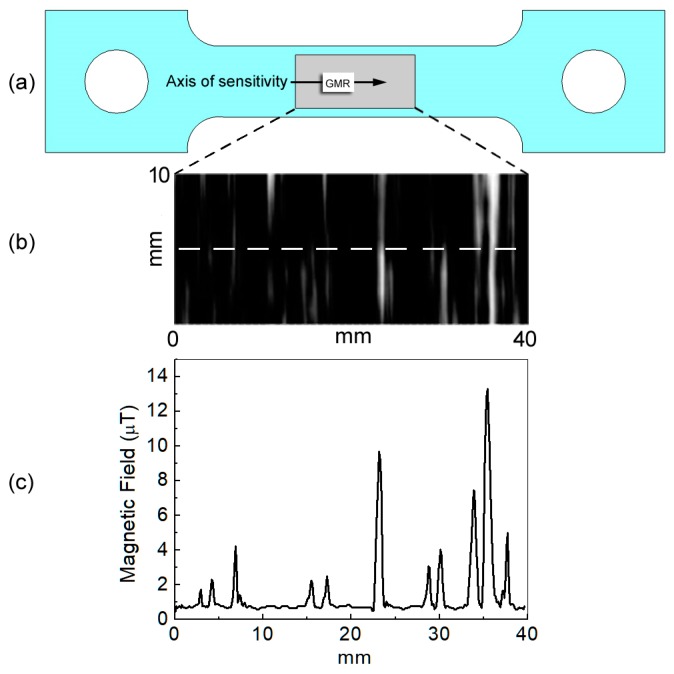
(**a**) Drawing of the sample where the rectangular grey area represents the area where the GMR gradiometer imaging has been obtained. (**b**) GMR imaging of the sample surface, the white stripes are due to plastic deformation (slip bands). (**c**) Magnetic field measured along the white dashed line reported in (b).

Moreover, as mentioned before, it is important to detect the plastic deformation by the time a crack failure occurs. This means that it is useful to localize the nucleation areas into the sample by using the magnetic field variation imaging technique. Then, by scanning the sample surface, it is possible to record a magnetic map of the gradiometer signal that highlights the magnetic dislocation, as shown in Figure 11. 

The magnetic image was obtained using 2nd order GMR gradiometer scanning on a small area of the test sampling, 10 mm × 40 mm (the grey rectangular area reported in Figure 11a), after a load cycle up to 40 kN. In the picture shown in Figure 11b, the white stripes that are clearly visible represent the slip bands due to the plastic deformation. The spatial resolution is 50 μm, and the magnetic field ranges from 0.4 μT (black colour) to 14 μT (white colour).

The magnetic field shows the slip bands, generally called persistent slip marking (PSMs), that appear on the structure surface by means of intrusions. Typically, the slip bands come prior to the initiation of the fatigue cracks after plastic deformation. The magnetic field reported in Figure 11b detects the presence of the slip marking caused by the magnetic dislocation density. The GMR inspection, due to its high sensitivity to low magnetic fields and its good spatial resolution, allows the detection and localization of the band position. Unless a nucleation occurs, the slip bands are not visible on the sample surface. For this reason, the enhancement offered by this method with respect to the conventional techniques consists in revealing, in time, the effect of the stress loading changes due to the sensitivity of the gradiometer sensor. 

## 5. Conclusions

In this work, a non-destructive evaluation technique to localize fatigue effects in the structural steel sample Fe360 by using the magnetic signal is reported. The results demonstrate the relationship between the microstructural changes induced by the applied strain and the associated magnetic field variations detected nearby the surface of the ferromagnetic specimens.

A tensile testing machine, made of non-magnetic material, was realized to stress the specimens. Moreover, an experimental set up was developed to record and process the experimental data. The experimental results, obtained using the 2nd order electronic gradiometer based on GMR on-chip sensors, suggest an unconventional method to localize the effect of the fatigue by means of magnetic signal variation. 

Finally, the reported technique allows the identification of the tensile concentration due to the magneto-mechanical effect before the crack initiation during the tensile load. This information could be useful for the study of the mechanical properties of the large metallic structures during their life cycle.
